# Comparative High-Resolution Transcriptome Sequencing of Lymphoma Cell Lines and *de novo* Lymphomas Reveals Cell-Line-Specific Pathway Dysregulation

**DOI:** 10.1038/s41598-018-23207-7

**Published:** 2018-04-19

**Authors:** Leila Taher, Julia Beck, Wen Liu, Catrin Roolf, Jan T. Soller, Barbara C. Rütgen, Sabine E. Hammer, Murali Chodisetti, Sina Sender, Katharina A. Sterenczak, Georg Fuellen, Christian Junghanss, Bertram Brenig, Ingo Nolte, Ekkehard Schütz, Hugo Murua Escobar

**Affiliations:** 1 Bioinformatics, Department of Biology, Friedrich-Alexander-Universität Erlangen-Nürnberg, Erlangen, Germany; 2 Institute for Biostatistics and Informatics in Medicine and Ageing Research, Rostock University Medical Center, Rostock, Germany; 3 Chronix Biomedical Göttingen, Göttingen, Germany; 4 Division of Medicine, Hematology, Oncology and Palliative Medicine, University of Rostock, Rostock, Germany; 5 Small Animal Clinic, University of Veterinary Medicine Hannover, Hannover, Germany; 6 Institute of Veterinary Medicine, University of Goettingen, Göttingen, Germany; 7 Clinical Pathology, Department of Pathobiology, University of Veterinary Medicine Vienna, Vienna, Austria; 8 Institute of Immunology, Department of Pathobiology, University of Veterinary Medicine Vienna, Vienna, Austria

## Abstract

In dogs as well as humans, lymphoma is one of the most common hematopoietic malignancies. Furthermore, due to its characteristics, canine lymphoma is recognized as a clinically relevant *in vivo* model to study the corresponding human disease. Immortalized cell lines are widely used as *in vitro* models to evaluate novel therapeutic agents and characterize their molecular mechanisms. However, it is known that long-term cultivation leads to clonal selection, genetic instability, and loss of the initial heterogenic character, limiting the usefulness of cell lines as preclinical models. Herein, we present a systematic characterization and comparison of the transcriptomic landscape of canine primary B- and T-cell lymphomas, five lymphoid cell lines (CLBL-1, CLBL-1M, GL-1, CL-1, and OSW) and four non-neoplastic control samples. We found that lymphomas and cell lines exhibit a common “differentiation and proliferation signature”. However, our analysis also showed that, independently of the cell of origin, the transcriptional signatures of lymphomas are more similar to each other than they are to those of cell lines. In particular, we observed that not all common therapeutic targets are similarly expressed between lymphomas and lymphoid cell lines, and provide evidence that different lymphoid cell-lines should be used to model distinct aspects of lymphoma dysregulation.

## Introduction

Lymphoma is one of the most common hematopoietic malignancies. Approximately 89% of human lymphomas are non-Hodgkin’s lymphomas (NHLs), and they represent about 5% of all newly diagnosed cancer cases per year in the United States (SEER Cancer Statistics Factsheets^1^). Similarly to human NHLs, canine lymphomas (CLs) account for 83% of all hematopoietic malignancies, and approximately 6% of all canine neoplasias^2,3^. Additionally, there is a remarkable resemblance between CLs and NHLs, including clinical presentation, tumor biology, and response to therapeutic agents, such as conventional chemotherapy. Akin to their human counterparts, CLs are classified into several subgroups, with B- and T-cell lymphomas being the most prominent. B-cell lymphomas represent approximately 70% of the reported cases, while T-cell lymphomas are seen in around 20%^4^. Equally important, lymphomas occur naturally in dogs in the presence of an active immune system. Further, dog physiology and drug metabolism are more similar to those of humans than of rodents, making CL an invaluable model for NHL research.

The usual therapeutic management of CLs is similar to that of NHLs and consists of a CHOP (Cyclophosphamide, Hydroxydaunorubicin, Oncovin, and Prednisone)-based chemotherapy. A complete remission is habitually achieved within three weeks, but the majority of canine patients relapse within the following twelve months. Therapeutic response and survival are highly correlated with lymphoma subtype, which is normally determined by immunophenotypic analysis^5–7^. Current routine lymphoma diagnosis and classification largely depends on cytologic evaluation, flow cytometric phenotyping and histopathological WHO classification, and, occasionally, PCR for antigen receptor gene rearrangement (PARR). In canine patients, advanced directed therapeutic interventions is hampered by limited detailed information on molecular markers for dysregulated pathways, tumor character, and consequential chemotherapeutic responsiveness and tolerance.

Immortalized cancer cell lines are frequently used as *in vitro* cancer models. Indeed, cell lines permit measurements under controlled conditions, and novel drugs and drug combinations are primarily evaluated in suitable cell line sets. Moreover, long-term cultivation of primary lymphoma material is infeasible. Hence, lymphoma therapy response at phenotypic, cytomorphologic, and other molecular levels are studied in lymphoid cell lines, which are widely accepted as a relatively stable *in vitro* system for these purposes. Commonly used CL cell lines include CLBL-1, CLBL-1M, GL-1, CL-1 and OSW^8–10^. In particular, CLBL-1 represents a canine B-cell lymphoma cell line comprehensively characterized and compared to primary neoplastic samples^11–14^. Nevertheless, despite the indisputable value of cell lines, clonal selection and long-term passages *in vitro* are known to lead to the loss of many attributes of the initial tumor, e.g. its heterogeneity^15^, and thus, well established cancer cell lines often show distinctive expression profiles from their clinical counterparts^16^. A comprehensive comparative molecular analysis of lymphoid cell lines and primary lymphomas is crucial to identify concordant and discordant biological functions, processes, and/or pathways. Additionally, this knowledge should enhance the efficiency of drug screening.

High-throughput gene expression analysis has proven to bear major potential in human^17–20^ as well as in veterinary oncology^21–26^. Microarray-based analyses of different CL subtypes allowed Frantz *et al*. (2012) to define three major prognostically significant groups and propose a robust real-time PCR-based screening assay that relies only on four genes (*CD28*, *ABCA5*, *CCDC3* and *SMOC2*). Furthermore, comparative microarray-based studies in canine and human diffuse large B-cell lymphomas (DLBCL) revealed the activation of the NF-KB pathway^24,25^ as well as the dysregulation of pathways potentially bearing therapeutic and prognostic value, such as the PI3K/AKT, Notch and JAK/STAT pathways^24^, in both species. Gene profiling has also found noticeable differences between human and canine lymphomas. Thus, in contrast to human B-cell lymphomas, canine B-cell lymphomas have been shown to rarely express *BCL6* and *MUM1/IRF4*^25^. Compared to conventional molecular approaches, massive parallel sequencing technologies offer numerous advantages: (i) they are not limited to known transcripts; (ii) they have a broader dynamic range of expression levels; (iii) they are more specific and sensitive; and (iv) can be used in a variety of applications, including the detection of transcript fusions, differential exon usage, and alternative splicing^27^. In particular, we have recently used mass parallel sequencing in order to characterize and evaluate experimental drug modes of action in the CL cell lines CLBL-1 and CLBL-1M as a model for human neoplasias^11^. Here, we comparatively characterize the transcriptional signatures of 16 primary CL samples, five CL cell lines and four non-neoplastic control samples by massive parallel sequencing (RNA-seq).

## Results

### The transcriptomic landscape of canine lymphomas and lymphoid cell lines

**Figure 1:**
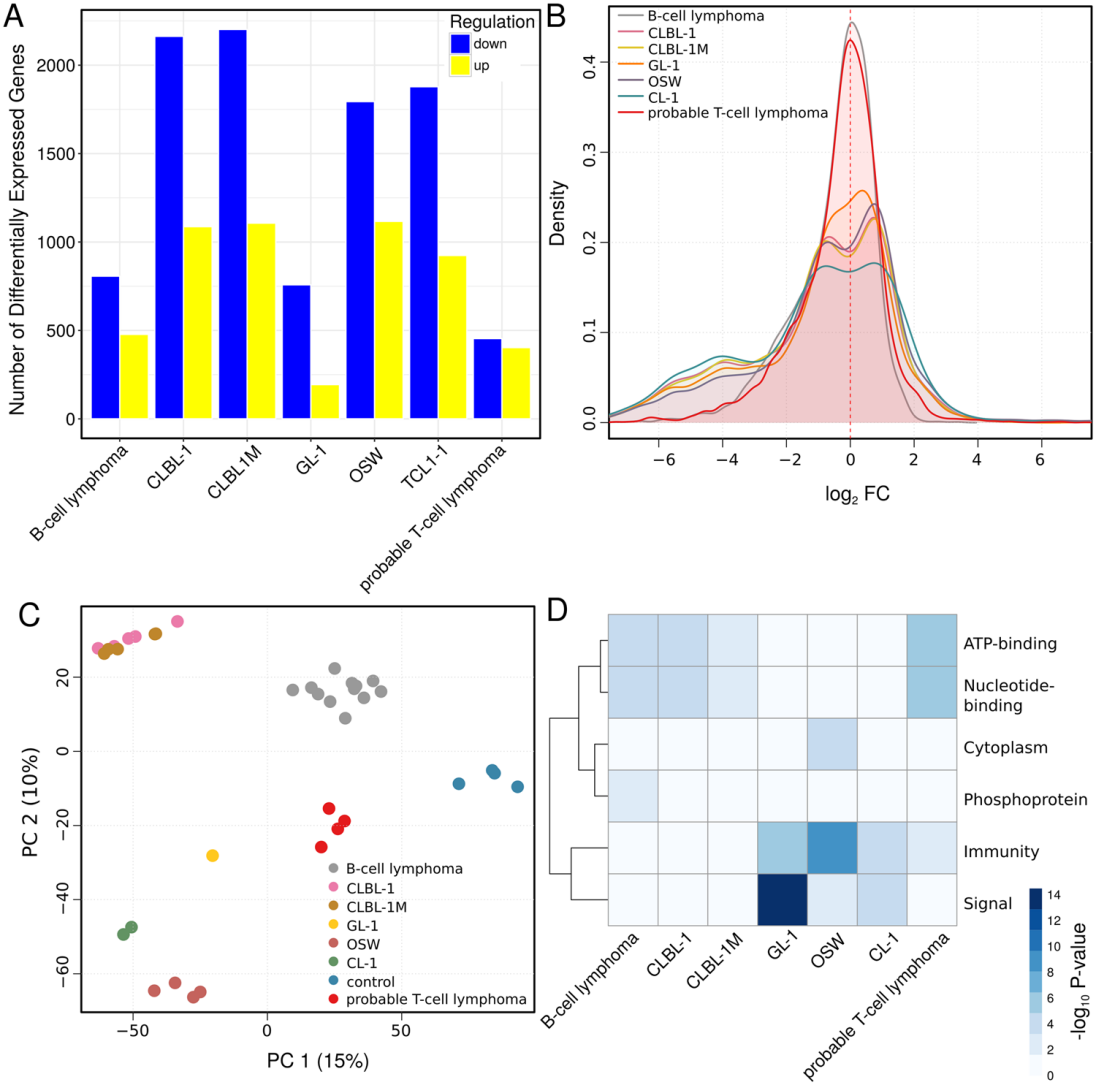
Significantly differentially expressed genes in the seven lymphoma and lymphoid cell line sample groups (B-cell lymphoma, probable T-cell lymphoma, CLBL-1, CLBL-1M, GL-1, OSW, and CL-1) compared to controls. (**A**) Barplot depicting the number of the significantly differentially expressed genes (DEGs) between each sample group and controls. (**B**) Distribution of logarithm (base 2) of the fold changes of the (regularized-logarithm) transformed expression values generated with the “DESeq2” R/Bioconductor package^52^ relative to controls. (**C**) Principal component analysis (PCA, the two first components are plotted) for (regularized-logarithm) transformed expression values generated with the “DESeq2” R/Bioconductor package. PC analysis was performed using the R function prcomp, on centered and scaled data. The first PC explains 15% of the variance in the data, the second PC, 10%. (**D**) UniProt keywords significantly associated with genes that are significantly differentially expressed in each sample group compared to controls. The Database for Annotation, Visualization and Integrated Discovery^57,58^ (DAVID Bioinformatics Resources 6.8, http://david.abcc.ncifcrf.gov/) was used to test for statistical enrichment of a given UniProt keyword at a FDR ≤ 5% with the set of 17,950 genes represented in the dataset with at least one sequence read count as background. Two-way clustering was performed using the Euclidean distance metric using complete linkage for both columns and rows. Visualization was performed with the R/Bioconductor package pheatmap^62^. Heatmap shades represent the negative log_10_ of the adjusted P-value (FDR) of enrichment of a given gene set for association with the listed UniProt keyword.

To investigate how representative lymphoid cell lines are of lymphomas, we systematically compared the transcriptomic profiles among primary canine lymphomas, five canine lymphoid cell lines, and four non-neoplastic lymph nodes, which were used as controls (see Methods). Specifically, we considered three lymphoid cell lines derived from B-cells (CLBL-1, CLBL-1M, GL-1) and two cell lines derived from T-cells (CL-1 and OSW). Twelve samples were classified as B-cell lymphomas and one sample was classified as T-cell lymphoma using flow cytometry (see Methods). Three additional lymphoma samples presented an uncertain phenotype, but were identified herein as probable T-cell lymphomas based on their transcriptomic profiles (see Fig. S1). Accordingly, the T-cell lymphoma sample was grouped together with the three “probable T-cell lymphomas” for the purpose of further analysis. The two resulting groups of primary lymphoma samples and the five lymphoid cell lines defined a total of seven sample groups, each with a different number of replicates. A total of 5,712 differentially expressed genes were identified in the lymphomas and lymphoid cell lines relative to controls (see Methods, Fig. 1A and Table S1). Of these, 1,703 were differentially expressed in lymphomas and 5,443 were differentially expressed in lymphoid cell lines, with most single lymphoid cell lines exhibiting larger numbers of differentially expressed genes than lymphomas. Additionally, both for lymphomas and lymphoid cell lines, most differentially expressed genes were down-regulated (1,055 and 3,553, respectively). Nevertheless, the majority of changes in expression were modest, with most genes (between 85 and 95%, depending on the sample group) exhibiting moderated log_2_ fold-changes between −2 and 2 (see Fig. 1B and Methods). Moreover, while extremely (log_2_ fold-change ≥ 5) up-regulated genes were not associated with any specific functions, extremely down-regulated genes (log_2_ fold-change ≤ 5) were associated with lymphocyte differentiation and apoptosis, among others, evidencing impaired lymphocyte activity.

Principal component analysis (PCA) of the moderated log_2_-transformed expression values for differentially expressed genes revealed a clear separation of the different sample groups, with few exceptions (see Fig. 1C). Thus, CLBL-1 and CLBL-1M appear to be undistinguishable, reflecting the common origin of these cell lines. Indeed, the cell line CLBL-1M was established from a CLBL-1 induced tumor in mouse, and has been shown to maintain the cell and molecular characteristics of CLBL-1^13^. Consistent with this general overview of the samples, differentially expressed genes among distinct sample groups featured specific functional profiles (see Fig. 1D).

We identified a total of 9 differentially expressed annotated microRNA genes (miRNAs) in lymphomas and lymphoid cell lines relative to controls (see Methods). These miRNAs showed relatively small differences in expression, with moderated log_2_ fold-changes between −2.6 and 2.4 with respect to control, and no two samples groups exhibiting opposite differential expression trends. Nevertheless, these differences were enough to distinguish between the lymphomas and lymphoid cell lines (see Fig. S2 and Table S2). Specifically, three miRNAs were up-regulated in at least one of the sample groups, while six miRNAs were down-regulated. Likewise, we found 97 differentially expressed annotated long non-coding RNA genes (lncRNAs) in lymphomas and lymphoid cell lines compared to controls (see Methods). The expression profiles of lymphoma and lymphoid cell line samples were clearly distinct, and among them, OSW cell lines stood out because of their relatively high expression levels for ENSCAFG00000033802, ENSCAFG00000035265, ENSCAFG00000038320, ENSCAFG00000038492, and ENSCAFG00000040754, which were virtually not expressed in other samples (see Fig. S3). In addition, only nine out of the 28 lncRNAs that were differentially expressed in B-cell lymphomas were also differentially expressed in one or more of the remaining sample groups, indicating that these lncRNA are specific B-cell lymphoma markers (see Table S3). Despite the limited annotation of the canine genome, these results highlight the potential power of non-coding RNAs as markers for lymphoma diagnosis.

Overall, our results indicate that whole genome transcriptomics can be used to reliably subtype CLs.

### A core of common genes controls cell differentiation and proliferation in both lymphomas and lymphoid cell-line models

Most of the 5,712 differentially expressed genes are most likely downstream effectors, that is, genes at the bottom hierarchical layers of the perturbed transcriptional and signaling networks. 1,703 genes were differentially expressed in lymphomas relative to controls, with 439 of these genes being differentially expressed in both B-cell and probable T-cell lymphomas (see Fig. S4 and Table S1). The vast majority of these 439 genes were differentially expressed in the same direction, with 227 and 206 genes being consistently up- and down-regulated, respectively, in both B-cell and probable T-cell lymphomas. These genes were significantly enriched in functions such as chromosome segregation, cell division, DNA metabolic process, and DNA replication whose association with cancer is well established. Among the functions enriched among the 846 genes that were exclusively differentially expressed in B-cell lymphomas we found locomotion, cell adhesion, cell proliferation, cell differentiation, cell death, immune system process, and response to stimulus. Further, many of these functions were also enriched among the 418 genes that were exclusively differentially expressed in probable T-cell lymphomas, suggesting the use of different molecular mechanisms to specify the same general biological processes and functions. Moreover, promoter analysis of the genes that were exclusively differentially expressed in each of these three groups (see Methods) revealed that they are most likely regulated by many of the same transcription factors, including E2F4 and SP1 from the transforming growth factor-β (TGF-β) pathway. The TGF-β pathway is key in cancer development and progression^28^.

Eighty-four percent of the 1,703 genes that were differentially expressed in lymphomas relative to controls were also differentially expressed in at least one lymphoid cell line. Moreover, 95% (1,362) of these genes were consistently up- and down-regulated; specifically, 481 genes were up-regulated in lymphomas and either up-regulated or unchanged in all lymphoid cell lines, while 881 genes were down-regulated in lymphomas and either down-regulated or unchanged in all lymphoid cell lines (see Fig. S5). These results indicate a relatively large number of shared molecular functions between lymphomas and lymphoid cell lines. Only 166 genes were differentially expressed in all lymphomas and lymphoid cell line sample groups. Among them, 78 genes were up-regulated and 86 were down-regulated in all sample groups. The two genes *SHCBP1* and *FLT3* (ENSCAFG00000003704 and ENSCAFG00000006716) exhibited discordant differential expression: *SHCBP1* is down-regulated in GL-1 cell lines and up-regulated in all remaining sample groups, whereas *FLT3* is up-regulated in GL-1 cell lines and down-regulated in all remaining sample groups. While *FLT3* mutations are not directly associated with lymphomas, *FLT3* mutations are frequently observed in acute myeloid leukemia and considered a negative prognostic factor for this disease^29^. The 166 differentially expressed genes were associated with molecular functions and biological processes such as cell cycle, mitotic cell cycle and nuclear division, chromosome segregation, organelle fission, DNA replication, nuclear and cell division, cytoskeleton organization, nucleotide binding, and ATPase activity, among others (see Table S4), representing the active division of cancer cells. Their promoters were significantly enriched in motifs consistent with the binding sites of members of the MEF2 and Forkhead box transcription factor families (see Methods). Mutations in members of the MEF2 family have been associated with leukemia and lymphoma^30^. In particular, mutations in MEF2B, the most divergent and least studied protein of the MEF2 family, promote lymphoma development^31^. Deregulation of Forkhead box (Fox) proteins is known to have a crucial role in T-cell activity and B-cell development^32^.

Taken together, these observations indicate the existence of a common core differentiation and proliferation signature for lymphomas and lymphoid cell lines, as has been reported for other types of cancer^33^.

### Distinct lymphoid cell-lines model different aspects of lymphoma dysregulation

**Figure 2:**
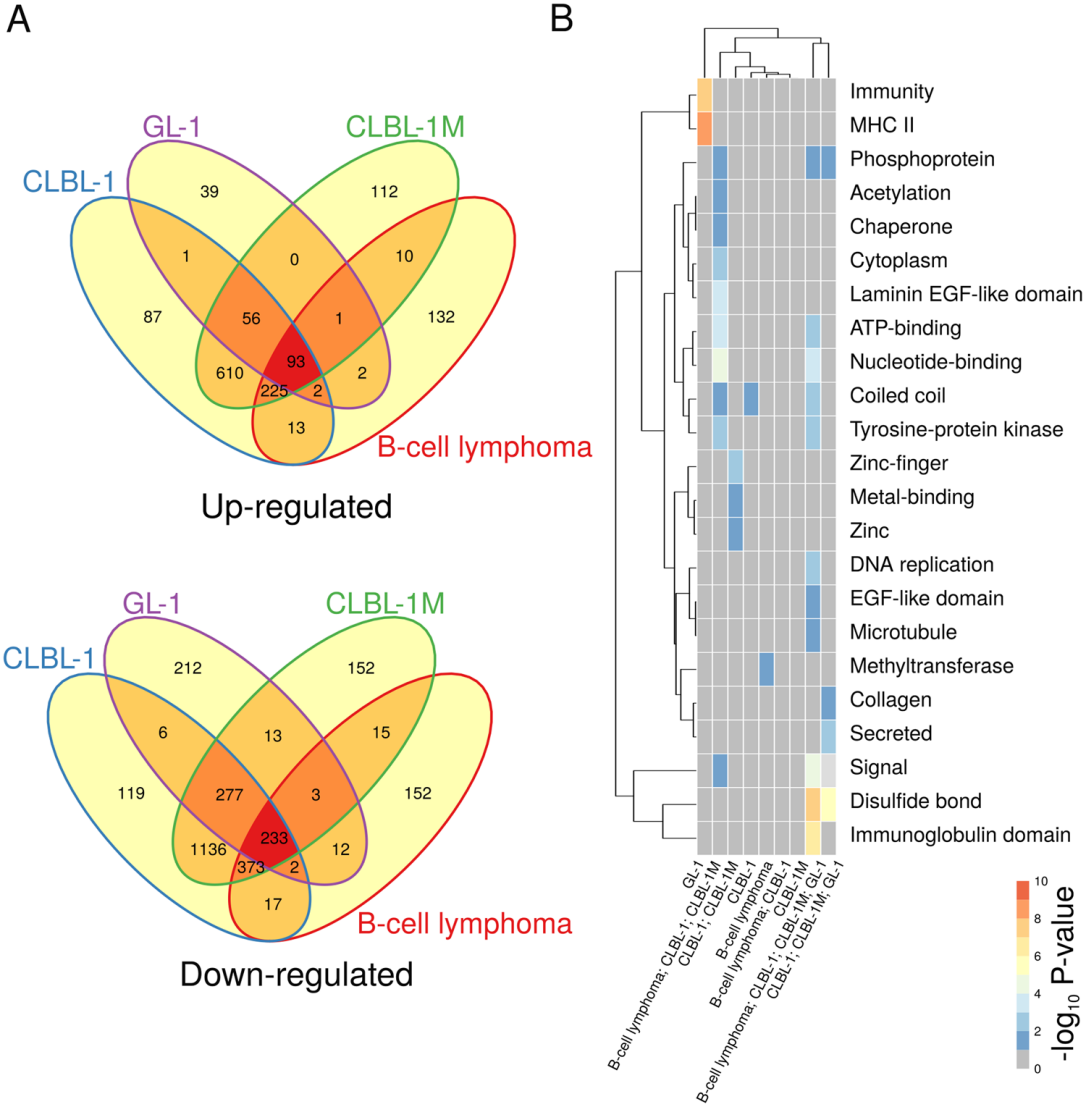
Comparison between B-cell lymphomas and B-cell-derived lymphoid cell-line models. (**A**) Venn diagram of consistently significantly up- and down-regulated genes in B-cell lymphoma and B-cell-derived lymphoid cell line samples. (**B**) UniProt keywords significantly associated with genes that are differentially expressed in B-cell lymphomas and/or B-cell-derived lymphoid cell lines. All considered sets are disjoint. For example, “B-cell lymphoma; CLBL-1” corresponds to the set of genes that are differentially expressed exclusively in both B-cell lymphoma and CLBL-1 samples. The Database for Annotation, Visualization and Integrated Discovery^57,58^ (DAVID Bioinformatics Resources 6.8, http://david.abcc.ncifcrf.gov/) was used to test for statistical enrichment of a given UniProt keyword at a FDR ≤ 5% with the set of 17,950 genes represented in the dataset with at least one sequence read count as background. Two-way clustering was performed using the Euclidean distance metric using complete linkage for both columns and rows. Visualization was performed with the R/Bioconductor package pheatmap^62^. Heatmap shades represent the negative log_10_ of the adjusted P-value (FDR) of enrichment of a given gene set for association with the listed UniProt keyword.

**Figure 3:**
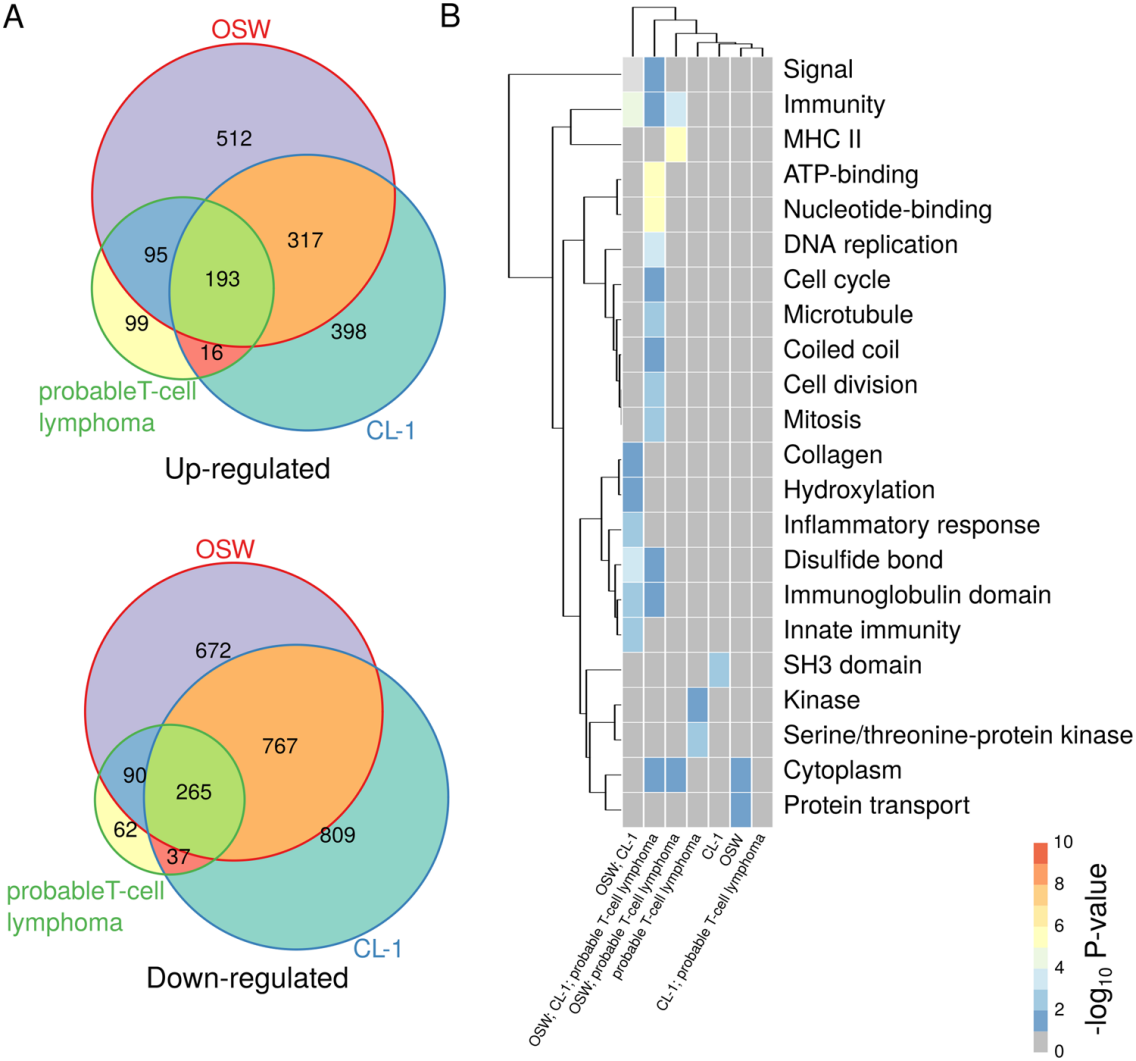
Comparison between probable T-cell lymphomas and T-cell-derived lymphoid cell-line models. (**A**) Venn diagram of consistently significantly up- and down-regulated genes in probable T-cell lymphoma and T-cell-derived lymphoid cell line samples. (**B**) UniProt keywords significantly associated with genes that are differentially expressed in probable T-cell lymphomas and/or T-cell-derived lymphoid cell lines (see Fig. 2).

Despite the relatively large overlap between the expression changes in lymphomas and lymphoid cell lines relative to controls, each lymphoid cell line exhibited a distinct expression profile. Thus, only 19% (93) of the genes that were up-regulated in B-cell lymphomas were also up-regulated in all B-cell-derived lymphoid cell lines (see Fig. 2A). Similarly, only 29% (233) of the genes that were down-regulated in B-cell lymphomas were also down-regulated in all B-cell-derived lymphoid cell lines. The products of the genes that were found differentially expressed in B-cell lymphomas and all B-cell-derived cell lines were enriched in kinases, proteins modified by the formation of disulfide bonds and containing coiled coil and EGF-like domains, immunoglobulins, and nucleotide-binding proteins (see Fig. 2B), and involved in cell development, proliferation and migration, among other functions (see Table S5). Moreover, the genes that were exclusively differentially expressed in each of the B-cell-derived sample groups (or closely related sample groups, in the case of CLBL-1 and CLBL-1M cell lines) exhibited distinct functional and molecular profiles (see Fig. 2B). Nevertheless, compared to other sample groups, the genes that were exclusively differentially expressed in B-lymphomas were only associated with relatively few specific processes or functions (see Fig. 2B and Table S5), suggesting few systematic differences in gene regulation and, therefore, strengthening the utility of lymphoid cell lines as lymphoma models. Analogously, 48% (193) of the genes that were up-regulated in probable T-cell lymphomas were also up-regulated in all T-cell-derived lymphoid cell lines, while 58% (265) of the genes that were significantly down-regulated in probable T-cell lymphomas were also down-regulated in all T-cell-derived lymphoid cell lines (see Fig. 3A). The products of the genes that were differentially expressed in all probable T-cell lymphoma and T-cell-derived cell line samples were found to be enriched in nucleotide-binding proteins, proteins with signal sequences and immunoglobulins, among others (Fig. 3B), and associated with cell proliferation, cell migration, and immune response, among others (see Table S6). Similarly as observed for B-cell lymphomas and B-cell-derived cell lines, the genes that were exclusively differentially expressed in probable T-cell lymphomas were associated with relatively few functions and processes (see Fig. 3B and Table S6).

**Figure 4:**
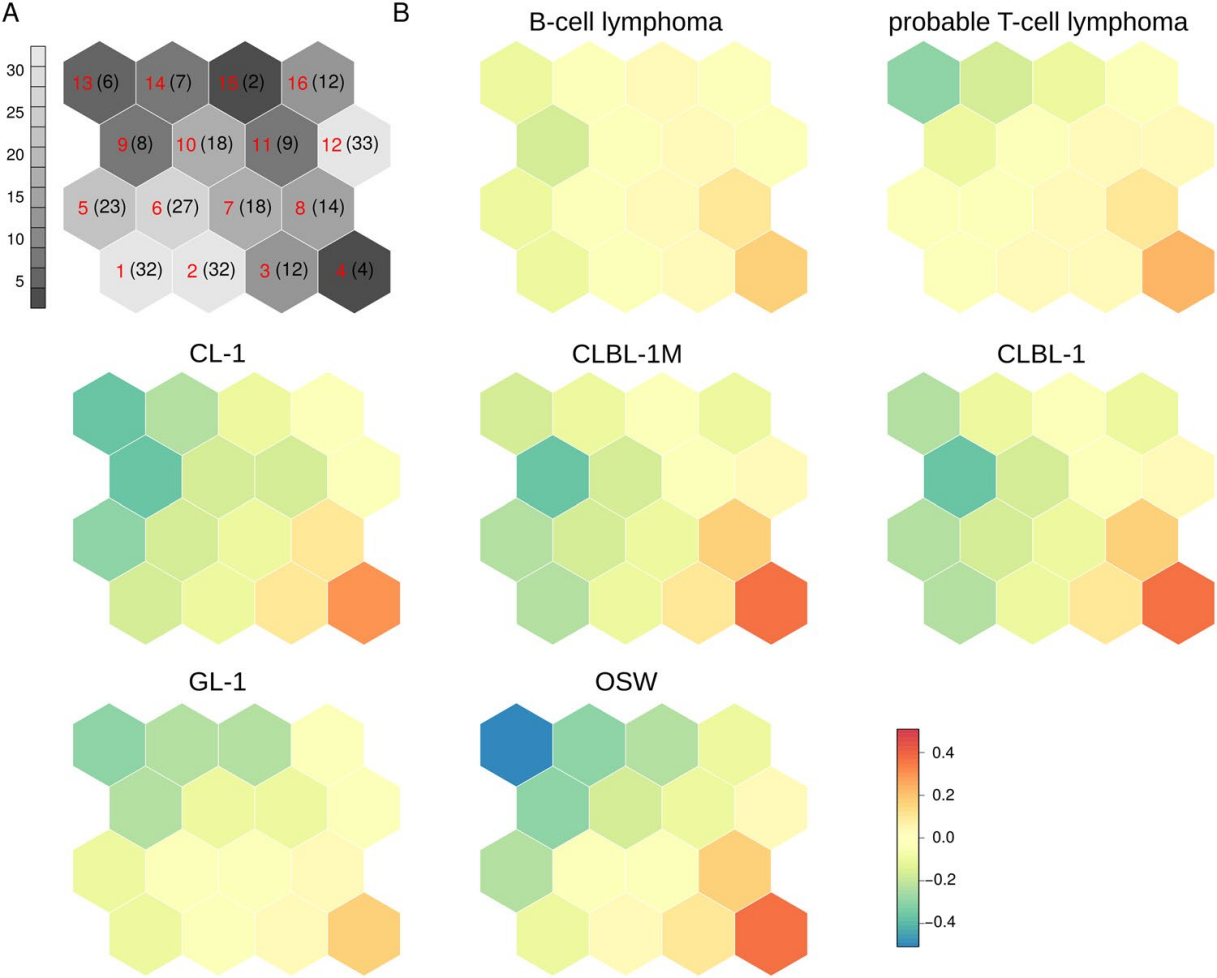
Self-organizing maps (SOMs) representing differences in pathway activity across sample groups. SOMs are a class of neural network algorithm for unsupervised machine learning with a strong visualization capability. Each hexagonal cell in the SOM grid is called a “unit” and represents a group of KEGG pathways^34–36^. Pathways have been grouped according to similarity in their “net expression” profiles across seven sample groups (B-cell lymphoma, probable T-cell lymphoma, CLBL-1, CLBL-1M, GL-1, OSW, and CL-1). The net expression of a pathway is defined as the number of significantly up-regulated genes minus the number of significantly down-regulated genes within each sample group in relation to the total number of genes in the pathway whose expression was detected by RNA-seq analysis (see Methods). (**A**) The gray scale and the numbers in parentheses indicate the number of pathways associated with each unit. The numbering of the units is indicated in red and corresponds to Table S7. (**B**) Each SOM map corresponds to a different sample group. The color of unit represents the average net expression of the corresponding group of pathways for the sample group indicated by the label of the SOM map, with blue indicating the lowest net expression and red the highest net expression. Two evident patterns are the red units in the right lower corner (units 4 and 8) and the blue units in the left upper corners (units 9 and 13). In contrast to lymphoid cell lines SOMs, lymphomas SOMs show only a few red and green units.

Genes that were differentially expressed in lymphomas or lymphoid cell lines were involved in virtually all examined KEGG pathways^34–36^ (257/260, see Methods). To identify common dysregulated pathways among the different sample groups we examined pathway net expressions, computed as the number of up-regulated minus the number of down-regulated genes within a sample group in relation to the total number of genes in a given pathway (see Methods). Net expression values varied between −0.58 (“tuberculosis”, in OSW cell lines, with 22 genes down-regulated out of 39) and 0.52 (“DNA replication”, in CLBL-1 cell lines, with 17 genes up-regulated and only one down-regulated out of 32), with a median of −0.06. In particular, several of the genes downregulated in the pathway with the most negative net expression values, “tuberculosis”, belong to the major histocompatibility complex class II (MHC II, 8) and/or are complement factors (11), which are key in immune response and antigen presentation. An impaired antigen presentation is essential for tumor cells to evade an immune response^37^. Consistent with the overall numbers of up- and down-regulated genes we found that a large number of pathways (between 122 and 205, depending on the sample group, with a median of 198) exhibited negative net expressions. Also, a total of 219 pathways had net expression values close to zero in at least one sample group, in the sense their absolute net expression values were in the range −0.05 to 0.05. Pathways with similar net expression profiles across all lymphoma and lymphoid cell line sample groups were clustered together using self-organizing maps (SOMs, see Fig. 4 and Table S7). The SOMs revealed that 28% (72) of the pathways had net expression values close to zero across most sample groups (units 7, 11, 12 and 16, with median net expression values across all pathways and sample groups in the range −0.05 to 0.05, see Fig. 4A and B). In addition, 37% (94) of the pathways exhibited negative net expressions in most sample groups (units 1, 5, 9, 10, 13 and 14, with median net expression values < −0.10), while only 7% (18) of the pathways had positive net expressions in most sample groups (units 4 and 8, with median net expression values > 0.10). Pathways with negative net expressions in lymphomas are those that would normally prevent tumor dissemination (e.g., “cell adhesion molecules” and “tight junction”) and that are required for establishing and/or maintaining the circadian rhythm and immune responses (e.g., “circadian rhythm” and “primary immunodeficiency”). In contrast, pathways with positive net expressions were associated with increased cell cycle (e.g., “DNA replication” and “pyrimidine metabolism”) and increased energy metabolism (e.g., “glycolysis gluconeogenesis” and “pentose phosphate pathway”). Furthermore, the SOMs indicated that pathway dysregulation is, in general, stronger in lymphoid cell lines compared to lymphomas. Indeed, of the aforementioned 94 pathways with negative net expressions in most sample groups (units 1, 5, 9, 10, 13 and 14), 31 had net expression values that were closer to zero in B-cell lymphomas than in any other sample group, while 56 had net expression values that were closer to zero in probable T-cell lymphomas than in any other sample group. Moreover, 81 pathways had net expression values that were closer to zero in B-cell or probable T-cell lymphomas compared to any other sample group. Similarly, as observed for pathways with negative expressions, of the aforementioned 18 pathways with positive net expressions in most sample groups (units 4 and 8), 4 had net expression values that were closer to zero in B-cell lymphomas than in any other sample group, while 2 had net expression values that were closer to zero in probable T-cell lymphomas than in any other sample group. Moreover, the only sample group which exhibited net expression values closer to zero than any of the lymphomas for more pathways was GL-1 (for 13 pathways). Despite the similarities in pathway dysregulation between lymphomas and lymphoid cell lines, we found punctual differences. For example, all lymphoid cell lines exhibited more down-regulated than up-regulated genes in the p53 signaling pathway (net expressions between −0.02 and −0.09), while B-cell and probable T-cell lymphomas displayed the opposite trend (net expressions 0.16 and 0.09, respectively). Inactivation or functional alternation of p53 by genomic loss or modulating mutations are frequently observed in human hematopoietic neoplasias including DLBCLs. Thereby, loss or mutation of p53 correlates with chemo resistance, shorter overall as well as progression free survival^38^. Consequently, the herein observed downregulation in the cell lines indicated a selection of an aggressive clone within the cell line compared to the primary samples. Consequently, if used for drug evaluation studies the analyzed cell lines are likely to represent chemotherapy-resistant cell fractions.

Overall, our data suggest that gene expression changes arising in *in vitro* culture affect multiple functions, processes, and pathways that are also affected in lymphomas. In turn, most if not all functions, processes, and pathways affected in B-cell lymphomas are altered in at least one of the lymphoid cell lines, while probable T-cell lymphomas appear to have a distinct expression profile. In general, we observed that most common pathways associated with cancer, and, in particular, lymphoma, are more profoundly dysregulated in the cell lines when compared to lymphomas, but the level of dysregulation and the identity of the pathways depends on the specific cell line.

### Not all pathway-specific drug targets in the B-Cell receptor signaling pathway are similarly expressed between lymphomas and lymphoid cell lines

**Figure 5:**
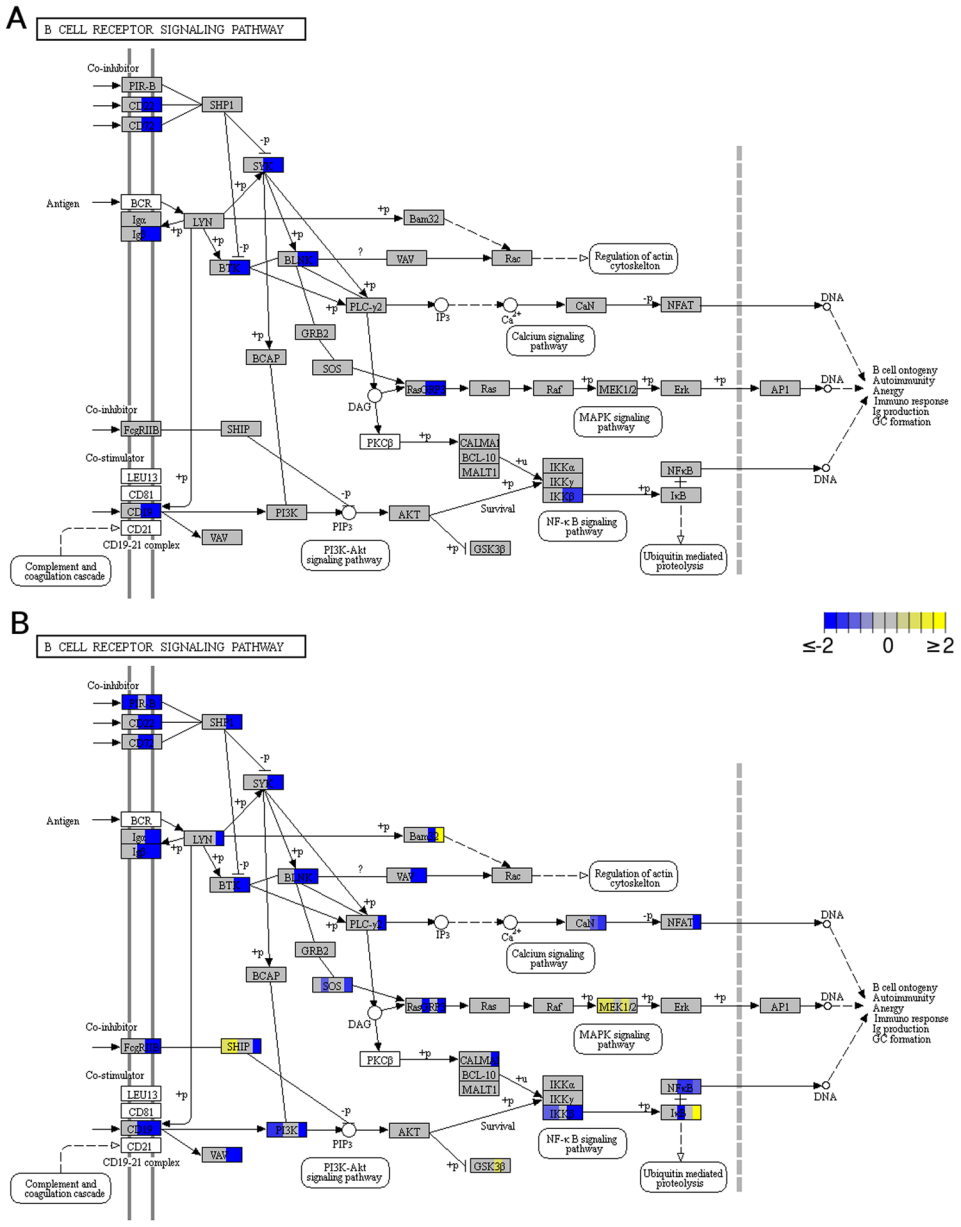
Pathway analysis of genes differentially expressed in the canine B-cell receptor signaling pathway (“cfa04662”) in the (**A**) lymphoma and (**B**) lymphoid cell-line sample groups. The boxes representing the genes are uniformly divided by the number of sample groups^34–36^. Sample groups are laid out from left to right as follows: (**A**) B-cell lymphoma and probable T-cell lymphoma, and (**B**) CLBL-1, CLBL-1M, GL-1, OSW, and CL-1. The logarithm in base 2 of the fold change relative to controls for the genes involved is indicated in yellow (up-regulated) or blue (down-regulated). White boxes represent genes whose expression was not detected. Fold changes were determined based on the average (regularized-logarithm) transformed expression values generated with the “DESeq2” R/Bioconductor package for each sample group^52^. The “Pathview” R/Bioconductor package^63^ was used to generate the graphical representation of the pathway and indicate which genes are differentially regulated.

Because of its centrality to hematopoietic neoplasias, many current human therapeutic approaches target proteins within the B-cell receptor signaling pathway. Indeed, several inhibitors are already in clinical use, FDA-approved or in clinical trials. In contrast to humans, dogs are usually not subjected to targeted therapeutic protocols, but the similarities in lymphoma presentation in both species suggest that pathway-specific inhibition should enhance treatment outcome in dogs as observed for humans. With the aim of evaluating the value of cell lines as *in vitro* models for pathway-specific inhibitor testing, we comparatively examined the expression of B-cell receptor pathway-inhibitor targets in the different lymphoid cell lines and primary lymphoma samples (see Fig. 5).

*GSK3B*, *AKT1*, *SYK*, *BTK*, and *PIK3CA* are common targets of kinase inhibitor therapies. Thus, *GSK3B* inhibitors are currently used to target different acute leukemia and lymphoma forms^39,40^. *AKT1* inhibitors are used to target different solid tumor types as well as leukemia and relapsed lymphoma forms^41^. Additionally, *SYK* has been shown to be frequently activated in primary human DLBCL^42^, and is a target for several human leukemia therapies. Thus, the *SYK*-inhibitor entospletinib is currently undergoing several clinical trials for hematopoietic malignancies. Likewise, BTK dysregulation has been implicated in a variety of B-cell-related diseases. Finally, four well-described *PI3K* isoforms (α, β, δ and γ) are encoded by *PIK3CA*, *PIK3CB*, *PIK3CD* and *PIK3CG*. Consequently, *PIK3* inhibitors are used in targeted therapeutic approaches for several human hematopoietic neoplasias and are in clinical trials for lymphoma types such as indolent non-Hodgkin’s lymphoma and DLBCL^43,44^. None of the aforementioned genes were differentially expressed in B-cell lymphomas relative to controls and most were not differentially expressed in probable T-cell lymphomas either. Only *SYK* was down-regulated (log_2_ fold-change −2.7) and *BTK* showed a borderline non-significant tendency towards down-regulation (log_2_ fold-change −2.0) in probable T-cell lymphomas. Among the cell lines, we found that *GSK3B* expression was slightly up-regulated in OSW cells (log_2_ fold-change 1.0), while *AKT1* was not differentially expressed. *SYK* and *BTK* were down-regulated in OSW and CL-1 cell lines (*SYK* had log_2_ fold-changes −5.0 and −5.0, respectively, and *BTK* had log_2_ fold-changes −4.2 and −4.2, respectively). Lastly, *PIK3CD* was down-regulated (log_2_ fold-change −3.2) in CL-1 cell lines. Independently of their efficacy as therapeutic agents in the canine model, *AKT1*, *SYK* and *BTK* expression is consistent between B-cell lymphomas and B-cell-derived cell lines as well as between probable T-cell lymphomas and T-cell-derived cell lines, indicating the suitability of the cell lines to evaluate AKT1, SYK and BTK inhibitors. In contrast, the divergence in expression observed for *GSK3B* and *PIK3CD* between lymphomas and cell lines is likely due to clonal selection or cultivation induced artifacts, hence limiting the value of the cell lines as drug testing system. It is important to note, though, that since kinase inhibitors show major effects in protein phosphorylation patterns, further evaluation at the protein level using canine specific antibodies should be warranted to evaluate the relevance of these findings.

## Discussion

Historically, classification of lymphomas has been dominated by histological and histopathological methods, but has recently evolved with the application of high-throughput transcriptomics and proteomics technologies. Thus, in humans, genome-wide gene expression profiling has led to the definition of different molecular subtypes with distinct prognosis^22^. Our results confirm that massive parallel sequencing (RNA-seq) can also be used for classification of B- and T-cell lymphomas in dogs, enabling a depth of analysis that was not feasible with conventional PARR (PCR for Antigen Receptor Rearrangement) and flow cytometry.

Cell lines are among the most used models in lymphoma studies^45,46^. Our comparative analyses of primary lymphoma and lymphoid cell line samples at the transcriptome level indicate that *in vitro* cultured lymphoid cell lines at least partially retain the transcriptomic landscapes of primary lymphomas. Specifically, we found that the majority of the differentially expressed genes are down-regulated compared to the controls. This suggests that an important mechanism in both lymphoid neoplasms and lymphoid cell lines involves turning off, rather than activating the expression of genes. Consistently, extremely down-regulated genes were highly enriched for normal lymphocyte functions, most likely indicating a dedifferentiation of the cells. In addition, genes that were differentially expressed in both primary lymphomas and lymphoid cell lines relative to controls were strongly associated with cell differentiation and proliferation. Distinct examples of proliferation signatures have been identified in many different tumor and tumor-derived cell types^33^. Basically, such proliferation signatures consist of a core set of genes whose expressions correlate with rapid cell proliferation and, they have been proposed as a component of clinical diagnostics for cancer patients, for example, to distinguish between low and high grade breast cancers^46^. We also observed a large number of genes whose expression was discordant between matched primary lymphomas and lymphoid cell lines. These alterations indicate cell population selection during primary and long-term culture. Moreover, the number of genes significantly differentially expressed in lymphoid cell line samples is many-fold larger than that in primary lymphoma samples. Many of these changes appear to represent a more extensive dysregulation of biological functions, processes, and/or pathways that were already dysregulated in the primary lymphomas. Despite the possibility of cross contamination of the primary tumors with non-neoplastic tissue, the fact that we found evidence for pathway- and cell-line-specific dysregulation suggests that *in vitro* culture leads to loss of tumor heterogeneity and clonal selection. Remarkably, we noted that not all common therapeutic targets in the B-cell receptor signaling pathway are similarly expressed between lymphomas and lymphoid cell lines, a finding that may have implications for the assessment of novel lymphoma drugs by cell line experiments and warrants further investigation.

In general, targeted therapies combining conventional chemotherapy with pathway-specific inhibitors improve treatment outcome. Although, in contrast to humans, dogs are normally not subjected to targeted therapeutic protocols, the similarities in lymphoma presentation in both species suggest that pathway-specific inhibition should enhance treatment outcome in dogs as well. Consequently, experimental therapeutic protocols in dogs bare significant value for human patients. Our systematic comparison between the transcriptome of primary lymphomas and lymphoid cell lines provides a solid rationale for the use of lymphoid cell lines as *in vitro* models for primary lymphomas, but also point out their limitations.

## Methods

### Cell lines and de novo samples

Canine B-cell lymphoma cell lines CLBL-1 (5 replicates), CLBL-1M (5 replicates), GL-1 (1 replicate), and canine T-cell lines CL-1 (2 replicates) and OSW (4 replicates) were cultured routinely in RPMI 1640//20% FBS/2% penicillin-streptomycin (all components from Biochrom AG) medium. All cells were maintained in a humidified incubator at 37 °C and 5% CO_2_.

Samples from dogs with naturally occurring lymphomas (12 multicentric B-cell lymphomas, one T-cell lymphoma, and three intestinal lymphomas) were collected from 16 patients at the Small Animal Clinic, University of Veterinary Medicine Hannover, Foundation, Germany according to the respective legislation of the state of Lower Saxony, Germany. Four non-neoplastic lymph nodes were used as controls.

### RNA isolation

Total RNA was isolated from primary lymphoma samples and lymphoid cell lines using the RNeasy Mini Kit (Qiagen) according to the manufacturer’s instructions. On-column DNase digestion was performed with the RNase-Free DNase Set (Qiagen) to avoid genomic DNA contamination. RNA quality was characterized by RIN analyses on an Agilent 2100 Bioanalyzer (Biorad, Germany) using the Nano Kit according to manufacturer’s instructions (Biorad, Germany).

### Library construction and sequencing

For the preparation of sequencing libraries, 2 µg total RNA with RIN > 8 were used. Poly-A RNA was enriched and ligated to sequencing adapter using the NEBNext Ultra RNA preparation Kit (New England Biolabs) according to manufacturer’s protocols. Sequencing was conducted on an Illumina Hiseq. 2500. 100bp-long paired-end reads were generated, with approximately 30 × 10^6^ reads per sample.

### Data processing and differential gene expression analysis

The reads were subsequently mapped to the canine genome (Broad CanFam3.1/canFam3, Sep. 2011) using BWA^47^. Duplicate reads and low quality mappings were removed using Picard (http://broadinstitute.github.io/picard) and samtools^48^. Raw read counts for each of the Ensembl^49^ (release 85, fpt://ftp.ensembl.org/pub/release-85/gtf/canis_familiaris) genes were obtained using the Python package HTSeq^50^. (version 0.6.1). Quality control was assessed using FastQC^51^.

Of the 19,856 annotated canine protein-coding genes in Ensembl, 17,950 genes (90.4%) were represented with at least one sequence read count (i.e., one mapped read) in at least 1 of the 37 individual sample RNA-seq libraries. These 17,950 genes were further filtered by removing lowly expressed genes, whereby only genes displaying a number of reads normalized by the library size above the 40th percentile for at least one sample were used for subsequent analyses, yielding 10,771 genes (54% of annotated canine genes). Of the 846 annotated canine microRNA genes (miRNAs) in Ensembl, 401 (48%) had at least one sequence read count (i.e., one mapped read) in at least 1 of the 37 individual sample RNA-seq libraries. These miRNAs were further filtered by removing lowly expressed miRNAs (below the 40th percentile for all samples), resulting in 246 miRNAs. Finally, of the 8,124 annotated canine long non-coding RNA genes (lncRNAs) in Ensembl, 4,729 (58%) had at least one sequence read count (i.e., one mapped read) in at least 1 of the 37 individual sample RNA-seq libraries. 1,889 where considered lowly expressed and excluded from the differential expression analysis, which included then a total of 2,840 lncRNAs.

The DESeq2 R/Bioconductor package^52^ (version 1.10.1) was used to process read counts from HTSeq and determine statistically differentially expressed genes. Our experimental design included cell lines and primary B- and T-cell lymphoma samples (B-cell lymphoma, probable T-cell lymphoma, CLBL-1, CLBL-1M, GL-1, OSW, and CL-1). Dispersion was estimated for these 10,771 genes using the function “estimateDispersions” with default options. The function rlogTransformation was used to transform the original count data to the log_2_ scale. For each of the primary lymphoma or cell line sample groups, the expression value of each gene was compared to the controls using pairwise comparison (Wald test with H_0_: moderated log_2_ fold-change = 0 and H_1_: moderated log_2_ fold-change ≠ 0). Genes were considered differentially expressed if the Benjamini-Hochberg adjusted P-value (false discovery rate, FDR^53^) was ≤1 × 10^−5^. The rlogTransformation() function in the DESeq2 R/Bioconductor package was used to transform the count data to the log_2_ scale; this transformation minimizes differences between samples for genes with small counts and normalizes the counts with respect to library size.

### Promoter motif analysis

We used DREME^54^ in conjunction with TOMTOM^55^ and JASPAR^56^ to identify putative transcription factor binding sites (TFBFs) in gene promoters. Gene promoters were defined as 1000-bp-long sequences starting 750 bp upstream and ending 250 bp downstream of the annotated transcription start site (TSS).

### Functional analysis

The Database for Annotation, Visualization and Integrated Discovery^57,58^ (DAVID Bioinformatics Resources 6.8, http://david.abcc.ncifcrf.gov/) was used to test for functional and pathway statistical enrichment analysis. In particular, we focused on the categories: GOTERM_MF_ALL, GOTERM_BP_ALL, GOTERM_CC_ALL, GOTERM_MF_DIRECT, GOTERM_MF_FAT, GOTERM_CC_FAT, UP_KEYWORDS, and KEGG_PATHWAY, and applied and identified enriched categories based on the FDR.

### Pathway analysis

Kohonen’s self-organizing maps (SOMs^59^) were used to extract prominent patterns of pathway activity. Specifically, SOMs were used to cluster and visualize the “net expression” of KEGG pathways^34–36^ across seven sample groups (B-cell lymphoma, probable T-cell lymphoma, CLBL-1, CLBL-1M, GL-1, OSW, and CL-1). The net expression of a pathway was defined as the number of significantly up-regulated genes minus the number of significantly down-regulated genes within each sample group in relation to the total number of genes in the pathway. Only genes whose expression was detected with at least one sequence read count in at least one RNA-seq library were considered. Definitions for 309 KEGG pathways were downloaded using the KEGGRest R/Bioconductor package^60^. The net expressions of 257 KEGG pathways comprising a minimum of 20 genes detected at the level of at least one sequence read count in at least one RNA-seq library were used to train the SOMs. Calculations were conducted with the kohonen R package^61^ with a 4 × 4 hexagonal SOM grid and additional default parameters.

The RNA-seq data presented in this manuscript has been deposited in the NCBI GEO database (entry GSE112474).

## Electronic supplementary material

Supplementary Figures and Legends

Supplementary Table 1

Supplementary Table 2

Supplementary Table 3

Supplementary Table 4

Supplementary Table 5

Supplementary Table 6

Supplementary Table 7
