# Supplementary material for: Comparative High-Resolution Transcriptome Sequencing of Lymphoma Cell Lines and de novo Lymphomas Reveals Cell-Line-Specific Pathway Dysregulation
Source: Sci Rep. 2018 Apr 19;8:6279. doi: 10.1038/s41598-018-23207-7 (PMC5908872; doi:10.1038/s41598-018-23207-7)
Supplement: Supplementary file 1 — Supplementary Figures and Legends [file 41598_2018_23207_MOESM1_ESM.docx]

**Comparative High-Resolution Transcriptome Sequencing of Lymphoma Cell Lines and de novo Lymphomas Reveals Cell-Line-Specific Pathway Dysregulation**

Leila Taher^1,2^, Julia Beck^3^, Wen Liu^4^, Catrin Roolf^4^, Murali Chodisetti^2,4^, Jan T. Soller^6^, Barbara C. Rütgen^7^, Sabine E. Hammer^8^, Sina Sender^4^, Katharina A. Sterenczak^4^, Georg Fuellen^2^, Christian Junghanss^4^, Bertram Brenig^6^, Ingo Nolte^5^, Ekkehard Schütz^3,6^, Hugo Murua Escobar^4,5 §^

^1^Bioinformatics, Department of Biology, Friedrich-Alexander-Universität Erlangen-Nürnberg, Germany

^2^Institute for Biostatistics and Informatics in Medicine and Ageing Research, Rostock University Medical Center, Rostock, Germany

^3^Chronix Biomedical Göttingen, Germany

^4^Division of Medicine, Hematology, Oncology and Palliative Medicine, University of Rostock, Rostock, Germany

^5^Small Animal Clinic, University of Veterinary Medicine Hannover, Hannover, Germany

^6^Institute of Veterinary Medicine, University of Göttingen, Göttingen, Germany

^7^Clinical Pathology, Department of Pathobiology, University of Veterinary Medicine Vienna, Vienna, Austria

^8^Institute of Immunology, Department of Pathobiology, University of Veterinary Medicine Vienna, Vienna, Austria

^§^ Corresponding author: Hugo Murua Escobar, Division of Medicine, Hematology, Oncology and Palliative Medicine University of Rostock, Ernst-Heydemann-Str. 6, 18057 Rostock, Germany, Tel: +49 381 4947519, Fax: +49 381 4945898.

Supplementary Figures

Figure S1


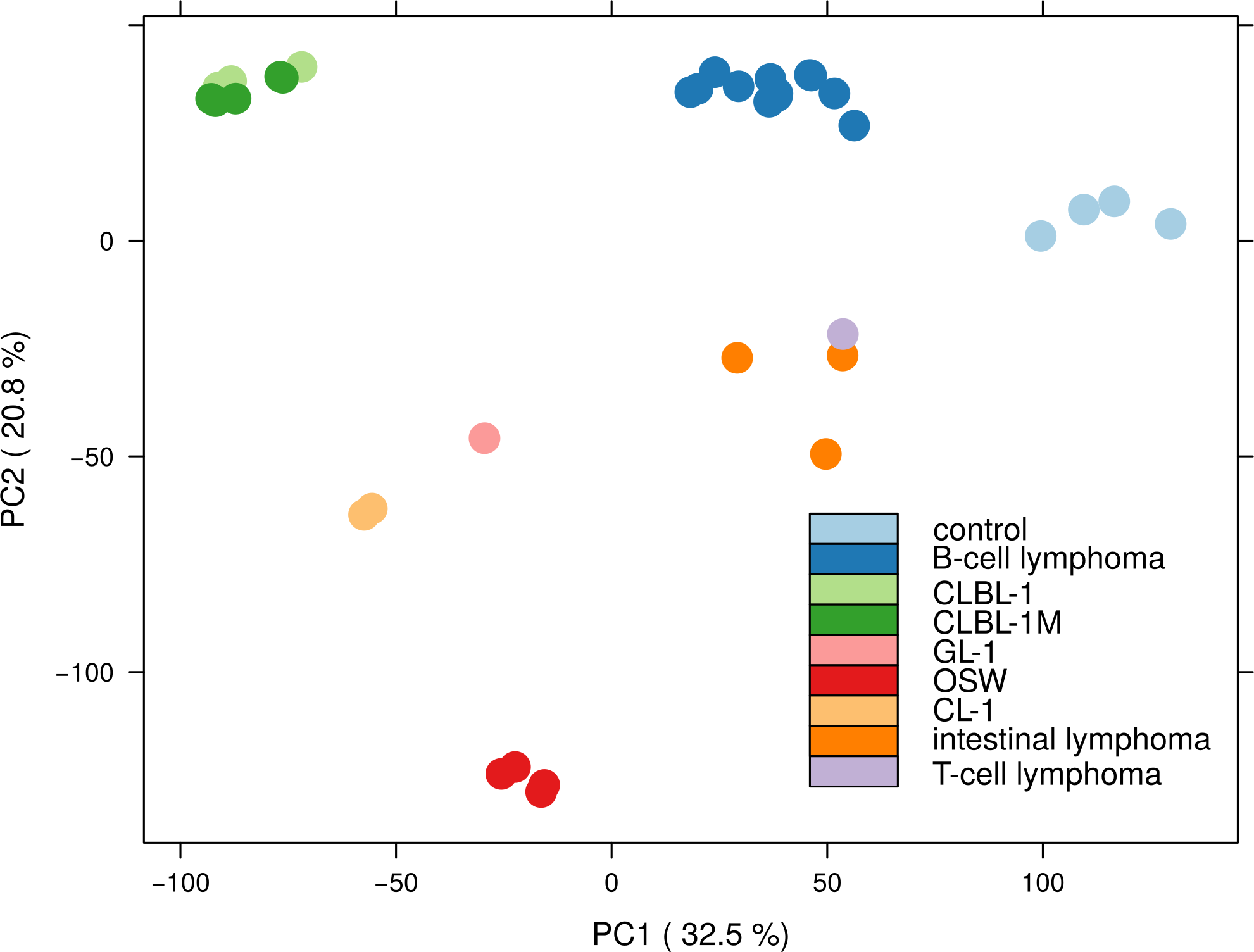


**S1 Fig. Principal component analysis (PCA, the two first components are plotted) for (regularized-logarithm) transformed expression values generated with the “DESeq2” R/Bioconductor package.** PC analysis was performed using the R function prcomp, on centered and scaled data. The first PC explains 322.5% of the variance in the data, the second PC, 20.8%. All 17,950 annotated protein-coding genes with at least one sequence read count (i.e., one mapped read) in at least 1 of the 37 individual sample RNA-seq libraries were included in the analysis. The single T-cell lymphoma sample clusters together with the three intestinal lymphoma samples. Because of the similarity between their molecular profiles, these four samples were further labeled as “probable T-cell lymphoma”.

Figure S2


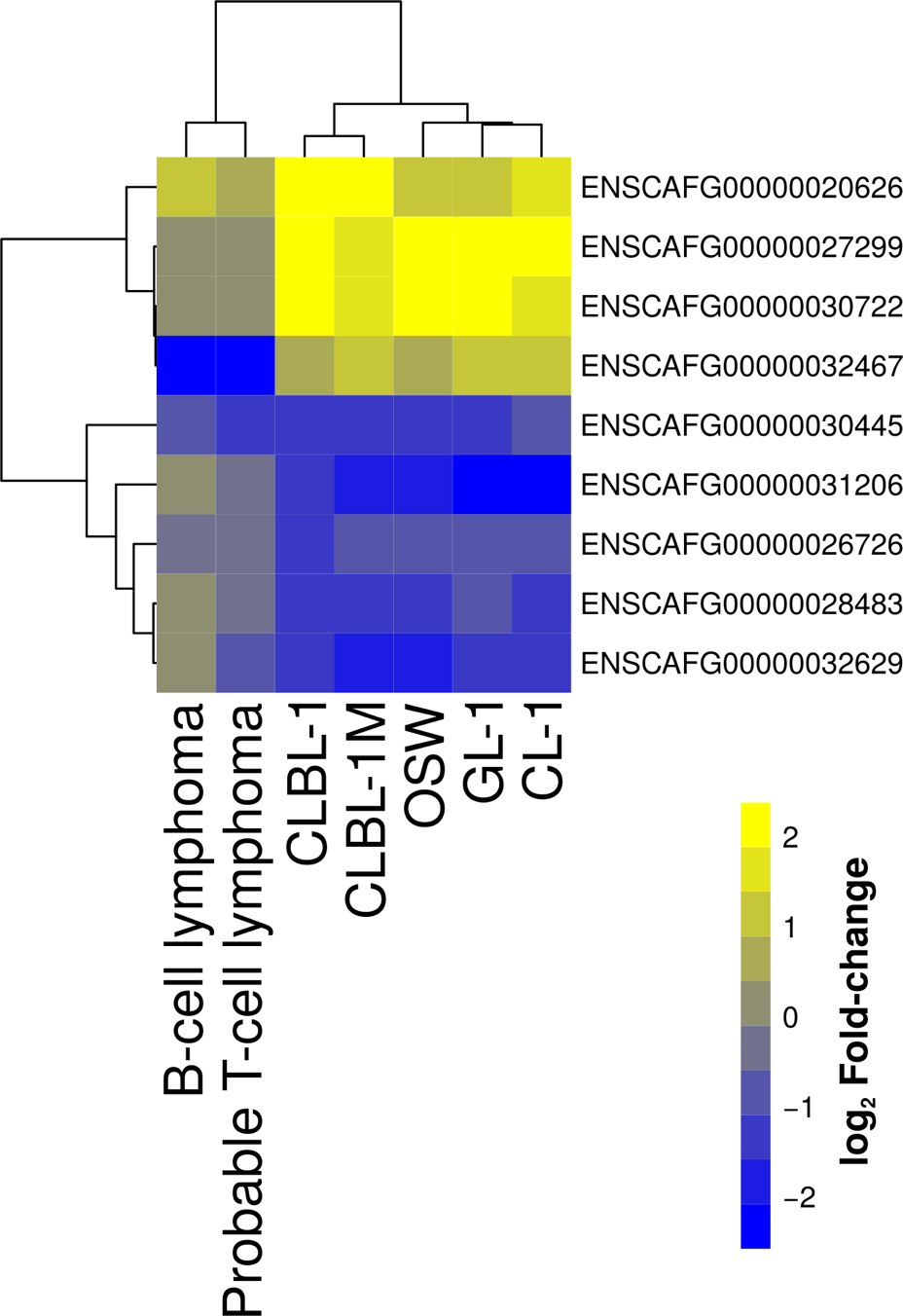


**S2 Fig. Heatmap showing log_2_ fold-changes of expression in differentially expressed miRNAs when compared to controls.** For each sample group, log fold-changes were calculated from average expression values. Such expression values had previously been subjected to DESeq2’s regularized-logarithm transformation. Two-way clustering was performed using the Euclidean distance metric for samples (columns) and a correlation-based metric for RNA transcripts (rows) using complete linkage for both sample and RNA transcripts tree construction. The heatmap was created with the “pheatmap” R/Bioconductor package ^1^.

Figure S3


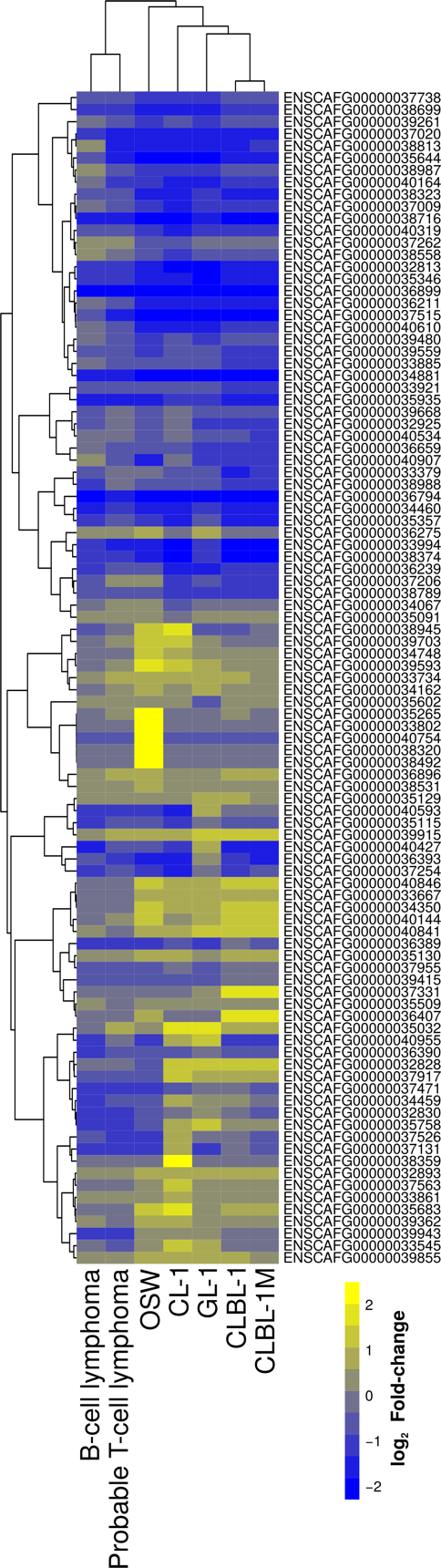


**S3 Fig. Heatmap showing log_2_ fold-changes of expression in differentially expressed lncRNAs when compared to controls.** (see S2 Fig).

Figure S4

**
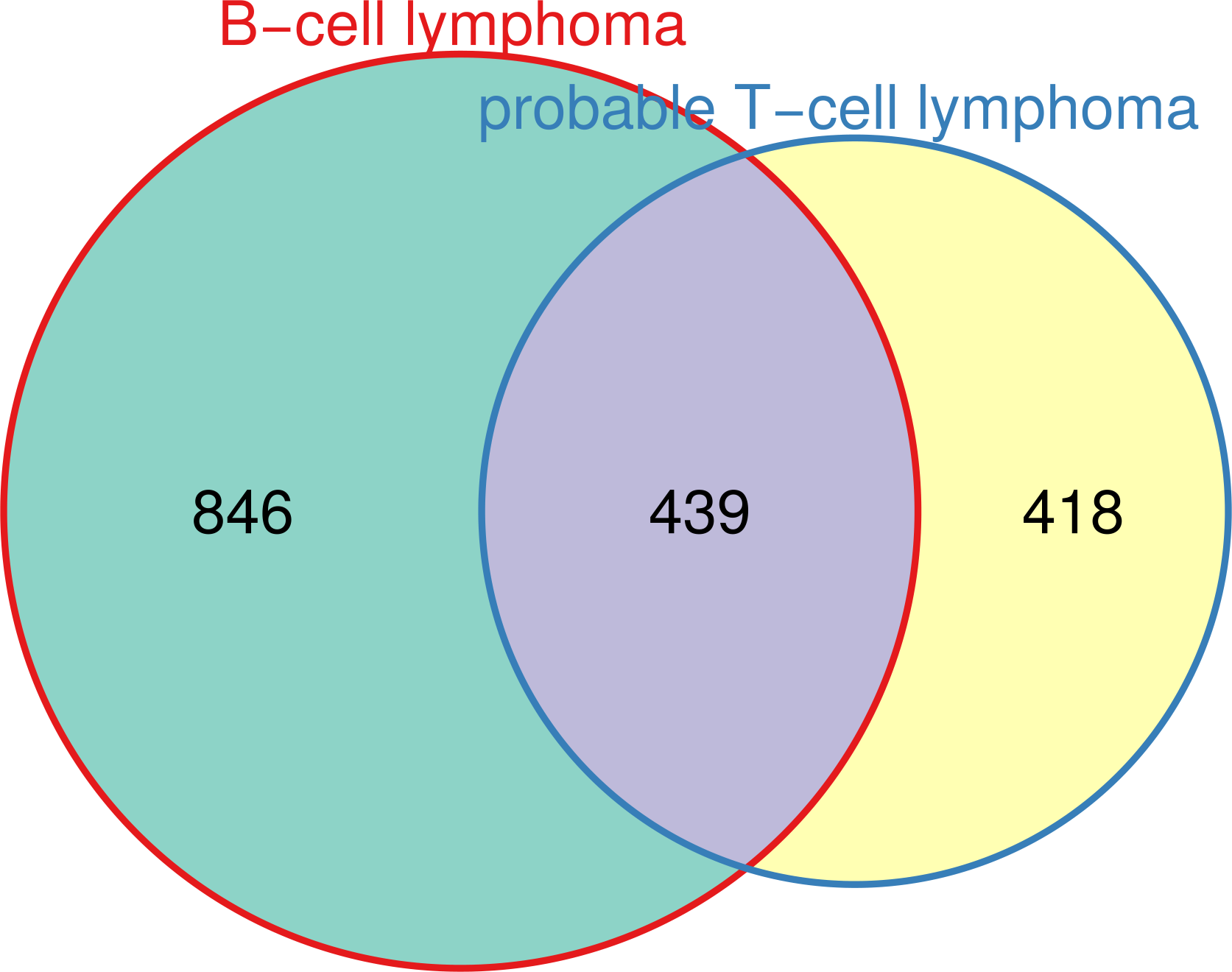
**

**S4 Fig. Venn diagram of the differentially expressed genes in B-cell and intestinal lymphomas relative to controls.** The number in each circle represents the amount of differentially expressed genes between B-cell lymphomas (red) and intestinal lymphoma (blue) as compared to controls. The intersection of the sets represents mutual differentially expressed genes.

Figure S5


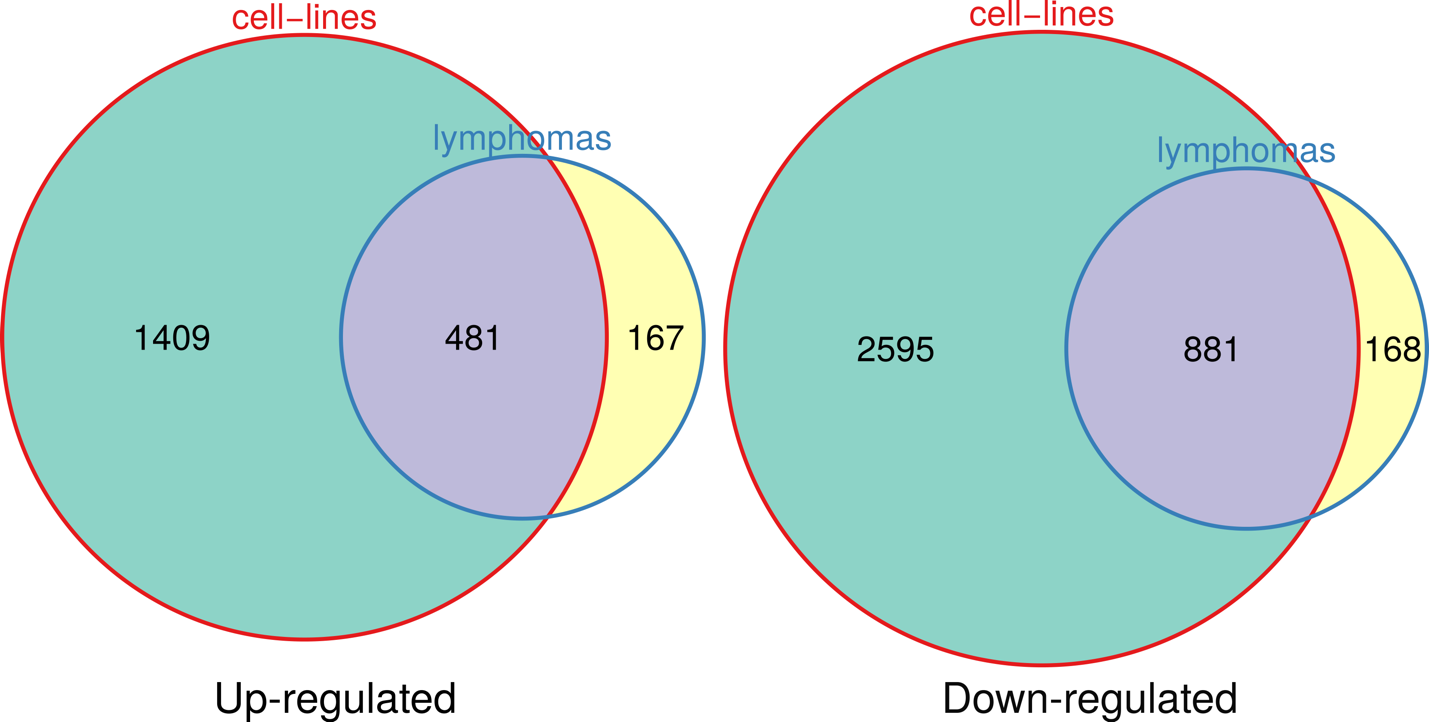


**S5 Fig. Comparison between lymphomas and lymphoid cell-line models.** (A) Venn diagram of consistently significantly up-regulated genes in lymphoma and lymphoid cell line samples. By “consistently up-regulated” we mean that the genes were up-regulated in B-cell and probable T-cell lymphomas and either up-regulated or unchanged in all lymphoid cell lines. (B) Venn diagram of consistently significantly down-regulated genes in lymphoma and lymphoid cell line samples. By “consistently down-regulated” we mean that the genes were down-regulated in B-cell and probable T-cell lymphomas and either down-regulated or unchanged in all lymphoid cell lines.

Supplementary Tables

**Table S1.** Ensembl gene id and symbol for the 5,712 protein-coding genes that were found significantly differentially expressed for at least one of the sample groups. The column “baseMean” is the mean of normalized counts of all samples, normalizing for sequencing depth. The column “Log2FoldChange” is the effect size estimate. It indicates changes in expression for a given sample group in comparison to controls. This value is reported on a logarithmic scale to base 2. The column “Padj”, or Benjamini-Hochberg-adjusted P-values, is the fraction of false positives (the false discovery rate, FDR) among the genes that are called differentially expressed for the corresponding P-value.

**Table S2.** Ensembl gene id and symbol for the 97 miRNAs that were found significantly differentially expressed for at least one of the sample groups (see S1 Table).

**Table S3.** Ensembl gene id and symbol for the 9 lncRNAs that were found significantly differentially expressed for at least one of the sample groups (see S1 Table).

**Table S4.** Functional analysis for enriched biological functions, processes and pathways related to the 166 genes which were significantly differentially expressed in all samples compared to controls. The background gene list used for this analysis was the list of all annotated canine protein-coding genes in the Ensembl database^2^ (release 85, fpt://ftp.ensembl.org/pub/release-85/gtf/canis_familiaris). The analysis was performed using DAVID^3,4^ (see Methods).

**Table S5.** Functional analysis for enriched biological functions, processes and pathways related to the 4,088 genes which were significantly differentially expressed in at least one B-cell lymphoma or B-cell-derived cell line sample compared to controls. The background gene list used for this analysis was the list of all annotated canine protein-coding genes in the Ensembl database (release 85, fpt://ftp.ensembl.org/pub/release-85/gtf/canis_familiaris, ^2^). The analysis was performed using DAVID (^3,4^, see Methods).

**Table S6.** Functional analysis for enriched biological functions, processes and pathways related to the 4,305 genes which were significantly differentially expressed in at least one probable T-cell lymphoma or T-cell-derived cell line sample compared to controls. The background gene list used for this analysis was the list of all annotated canine protein-coding genes in the Ensembl database^2^ (release 85, fpt://ftp.ensembl.org/pub/release-85/gtf/canis_familiaris). The analysis was performed using DAVID^3,4^ (see Methods).

**Table S7.** List of KEGG pathways associated with each unit in the self-organizing map (SOM). Pathway net expression values are indicated for each sample group (see Figure 4 and Methods).

**References**

1 pheatmap: Pretty Heatmaps v. version 1.0.8 (2015).

2 Yates, A. *et al.* Ensembl 2016. *Nucleic Acids Res* **44**, D710-716, doi:10.1093/nar/gkv1157 (2016).

3 Huang, D. W., Sherman, B. T. & Lempicki, R. A. Bioinformatics enrichment tools: paths toward the comprehensive functional analysis of large gene lists. *Nucleic Acids Res* **37**, 1-13, doi:10.1093/nar/gkn923 (2009).

4 Huang, D. W., Sherman, B. T. & Lempicki, R. A. Systematic and integrative analysis of large gene lists using DAVID bioinformatics resources. *Nat Protoc* **4**, 44-57, doi:10.1038/nprot.2008.211 (2009).
